# Hepato‐Renal Protective Potential of Dimethyl Fumarate in Alloxan‐Induced Diabetic Mice Model by Modulating of Sirt1, Nrf2 and Inflammatory Genes Expressions

**DOI:** 10.1002/edm2.70146

**Published:** 2026-02-06

**Authors:** Parisa Saberi‐Hasanabadi, Fatemeh Shaki, Mohammad Karami, Abouzar Bagheri, Mohammad Ranaee, Ramin Ataee

**Affiliations:** ^1^ Student Research Committee, Faculty of Pharmacy Mazandaran University of Medical Sciences Sari Iran; ^2^ Pharmaceutical Science Research Center, Hemoglobinopathy Institute Mazandaran University of Medical Sciences Sari Iran; ^3^ Department of Clinical Biochemistry and Medical Genetics, Faculty of Medicine, Immunogenetics Research Center Mazandaran University of Medical Sciences Sari Iran; ^4^ Clinical Research Development Unite of Rouhani Hospital Babol University of Medical Science Babol Iran; ^5^ Medicinal Plants Research Center Mazandaran University of Medical Sciences Sari Iran; ^6^ Thalassemia Research Center, Hemoglobinopathy Institute Mazandaran University of Medical Sciences Sari Iran

**Keywords:** alloxan, anti‐inflammatory responses, diabetes, dimethyl fumarate, hepato‐renal protective effects

## Abstract

**Aim:**

Despite advances in diabetes treatments, the effects of this disease have not yet been adequately reversed or prevented in patients. Therefore, development of more effective medication‐assisted treatments in this field is needed.

**Method:**

Type 1 diabetes mice models were established using multiple low‐dose alloxan, then were treated with three doses of dimethyl fumarate, that is, low, medium and high viz. 20, 40 and 80 mg/kg, respectively for 21 days. Then, specific tests were done to evaluate blood biochemical parameters, oxidative stress markers, inflammatory genes expression and histopathological changes in the mice kidneys and livers.

**Results:**

Improved anti‐diabetic, hepato‐renal protective and oxidative stress indexes of dimethyl fumarate in diabetic mice were shown. The histological features improved in comparison with diabetic mice. The real‐time PCR results indicated a decrease in the alloxan‐induced elevations in mRNA levels of pro‐inflammatory cytokines such as TNF‐α, IL‐6 and NF‐κB levels in both kidney and liver tissues of diabetic mice. Meanwhile, dimethyl fumarate showed an increase in Sirt1 and Nrf2 expression in comparison to the diabetic group.

**Conclusions:**

In total, it can be concluded that dimethyl fumarate treatment provides hepato‐renal protective effects on alloxan‐induced diabetic mice model by attenuating reactive oxygen species inflammatory pathways through modulating Sirt1/Nrf2 and inflammatory genes expressions. This study can be an introduction to further studies on the basis of diabetes treatment, especially clinical studies to demonstrate the effect of dimethyl fumarate in diabetes.

## Graphical abstract

Hepato‐renal protective potential of DMF in alloxan‐induced diabetic mice model.

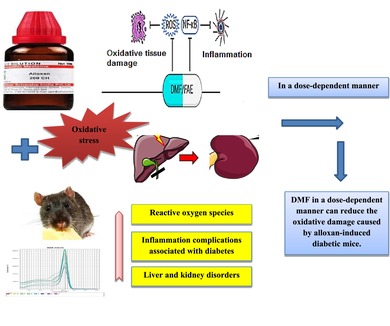

**Abbreviations**
AGEs: advanced glycation end‐products
ALT: alanine aminotransferase
AST: aspartate aminotransferase
BUN: blood urea nitrogen
CRT: creatinine
EDTA: ethylenediaminetetraacetic acid
GSH: glutathione
IL‐6: interleukin 6
MDA: malondialdehyde
MET: metformin
NF‐κB: nuclear factor‐κB
ROS: reactive oxygen species
Sirt1: sirtuin 1

## 1 Introduction

With the development of urbanisation in developing countries, we are witnessing an increase in the prevalence of diabetes [1]. Type 1 diabetes is a metabolic disorder of the carbohydrate cycle characterised by high blood glucose levels (hyperglycaemia) and is usually caused by insufficient production of the hormone insulin [2]. The general and rapid spread of this disease has caused destructive damage in many body organs, especially the liver and kidney [3]. Pathogenesis mechanisms in the early stages of this disease are unclear. Clear symptoms of this disease include increased tissue dysfunction and inflammation indicators [4, 5]. In this line, many studies have evaluated the relationship between liver abnormalities and diabetic nephropathy [3, 5, 6, 7]. The interaction of long‐term hyperglycaemia, hyperlipidaemia and hyperinsulinaemia can lead to several pathological responses, such as excessive formation of free radicals and increased activity of inflammatory cytokines. These significant changes eventually lead to liver fibrosis, nephropathy and ultimately changes in the structure and irreversible dysfunction of these two organs, which may be associated with side effects and high mortality [8]. Oxidative stress is considered the main prerequisite in the pathogenesis of diabetic nephropathy and liver cell damage. Biomarkers of oxidative stress such as glutathione levels, superoxide dismutase activity, advanced glycation end‐products (AGEs), the nicotinamide adenine dinucleotide phosphate (NADPH) oxidase activity, reactive oxygen species (ROS) and malondialdehyde (MDA) were reported to be altered in diabetic nephropathy and liver cell damage [9]. Increasing the antioxidant capacity and removing ROS is considered a promising strategy for the prevention and treatment of diabetic liver‐kidney dysfunction [10].

Dimethyl fumarate as a methyl ester of fumaric acid has been used to reduce cytokine and chemokine gene expression to treat psoriasis and multiple sclerosis by increasing anti‐inflammatory responses. The administration of dimethyl fumarate was similar to the effect of metformin (MET) in animal models by regulating the inflammatory pathway and improving oxidative markers along with antioxidant properties. As a modulator of the Nrf2 pathway and NF‐κB transmission, dimethyl fumarate reduces TNF‐α and manifests its antioxidant and anti‐inflammatory effects [11, 12]. Therefore, the ROS‐Sirt1, Nrf2 and inflammatory pathway might be a potential target for the prevention of hepato‐renal disorders in diabetic patients. However, until today, the protective effects of dimethyl fumarate on two organs affected by diabetes (liver and kidney) are not fully understood. Hence, the evaluation of this hypothesis shapes our present study. In previous studies, the potential mechanism of the protective effects of dimethyl fumarate on vascular complications from the ROS‐TXNIP‐NLRP3 inflammatory pathway was evaluated in diabetic rats. Dimethyl fumarate attenuates vascular remodelling and functional alterations in streptozotocin rats via several mechanisms. Based on these observations, dimethyl fumarate may be a promising drug for preventing vascular complications in diabetic patients possibly via impairing ROS‐TXNIP and/or ROS‐NF‐κB pathways [9].

The present study was designed to investigate the possible protective effects of dimethyl fumarate against a diabetic mice kidney and liver dysfunction model, in addition to analysing the role of inflammatory mediators, oxidative stress and blood biochemical indicators.

## 2 Materials and Methods

### 2.1 Chemicals

Alloxan was used to induce diabetic conditions in mice. MET was used as a drug control. Alloxan was purchased from Sigma Aldrich Chemical Co. (St. Louis, MO, USA). TRIzol reagent and MET were bought from Ramopharmin Pharmaceutical Co. (Tehran, Tran). Dimethyl fumarate was obtained from Tocris Neuramin (Bristol, UK). The kits for estimating levels of blood glucose, albumin and creatinine were purchased from Pars Azmoon Company (Tehran, Iran). All other chemicals were obtained from standard commercial suppliers. The chemicals used for conducting this research were of premium analytical quality and the chemical solutions were prepared fresh each time well before use.

### 2.2 Animal Treatments

Experiments were performed on 6–8 weeks female C57BL/6 mice (*n* = 35) weighting between 18.3 and 19.5 g. The animals were divided randomly into seven groups with five animals each. The number of mice and doses of the compounds used in this study were chosen in accordance with previous studies [4, 9, 10].

– Group I (G_I_)—Control group (normal saline).
– Group II (G_II_)—Alloxan (120 mg/kg/day) (Diabetic group).
– Group III (G_III_)—Alloxan + dimethyl fumarate (20 mg/kg/day).
– Group IV (G_IV_)—Alloxan + dimethyl fumarate (40 mg/kg/day).
– Group V (G_V_)—Alloxan + dimethyl fumarate (80 mg/kg/day).
– Group VI (G_VI_)—Alloxan + MET (200 mg/kg/day).
– Group VII (G_VII_)—Only dimethyl fumarate (80 mg/kg/day).

The mice were maintained at 22°C under a 12‐h light/dark cycle. Food and water were provided throughout the experimental period ad libitum. The animal experimentation protocols were conducted in accordance with the recommendations of the Mazandaran University of Medical Sciences Animal Ethical Committee (Code: IR.MAZUMS.4.REC.1401.11716). At the sacrifice (Day 21), mice were anaesthetised with Ketamine/Xylazine (40 mg/kg, ip) and their liver and kidney tissues were separated by laparotomy. First, the removed tissues were washed using cold mannitol buffer (including 0.255 M mannitol, 74 mM sucrose and 0.2 mM EDTA). The cut tissues were homogenised with an electric homogeniser. The homogenised tissue was transferred to microtubes and centrifuged at 2000 × *g* for 10 min in a refrigerated centrifuge at 4°C. After the centrifugation time, the supernatant solution was slowly transferred to other microtubes and the bottom sediment containing broken cells and nuclei was discarded. Then the supernatant solution was centrifuged again at 12,000 × *g* for 10 min. After 3 weeks of treatment, immediately before sacrifice, blood samples were collected by retro‐orbital puncture in flasks coated with 2 μl of EDTA (0.5 M) to prevent clotting. Under these conditions, plasma can be properly separated from blood by centrifugation (3000 × *g* for further 15 min) for biochemical measurements [9].

### 2.3 Diabetes Model and Treatment Methods

In current study, alloxan (120 mg/kg as a 5% solution in normal saline) was injected in single administration intraperitonially to the animals for inducing diabetes [13, 14]. The treatment was continued till 21 days; blood glucose level was measured on 21 days of post‐treatment. The mice were included in the study only if they were diabetic and had blood glucose level above 180 mg/dL.

### 2.4 Biochemical Analysis

Blood glucose, albumin, creatinine, urea, ALT and AST were determined with scientific kits available commercially. Sampling was done at a specific time. Blood urea and creatinine levels were measured using a special diagnostic kit (Pars Azmoun Company, Iran). In order to measure blood albumin, bromcresol green (BCG) dye‐binding method was used by pars azmoun bromocrosol kit. The blood glucose was determined by a commercial kit (Pars Azmoon Diagnostics, Iran) based on the colorimetric glucose oxidase method [15]

### 2.5 Analysis of Oxidative Stress Markers in Kidney and Liver Homogenate

Mice kidney and liver were washed and homogenised in mannitol buffer. The supernatant was collected in the first centrifuge (5000 × *g*), and sediments (in the second centrifuge stage; 11,000 × *g*) were distributed in tris buffer for evaluation of protein carbonyl, glutathione and MDA content. Glutathione level and MDA content were analysed spectrophotometrically, as described earlier [16]. Briefly, for evaluation of glutathione content, 5,5′‐dithiobis‐2‐nitrobenzoic acid (DTNB) (0.04%) was used (as an indicator), and the absorbance was calculated at 412 nm. The spectrophotometric assay using 2, 4‐dinitrophenylhydrazine constitutes one of the primary ways of detecting protein carbonyl content, since it is relatively easy, fast and inexpensive [17].

### 2.6 Histopathological Examination

In briefly, the tissue samples from the livers and kidneys (*n* = 3 organ samples for per group) were fixed in 10% neutral buffered formalin solution (pH 7.4), dehydrated in gradual ethanol (70%–100%), cleared in xylene and embedded in paraffin. Five‐micrometre sections were prepared and then routinely stained with haematoxylin and eosin (H&E) dyes [18]. Stained slides (75 × 25 mm) were microscopically analysed using light microscopy (BX40; OLYMPUS, Japan).

### 2.7 Quantitative Real‐Time RT‐PCR

Total RNAs were extracted from tissues using TRIzol reagent (YTA, Iran) and treated with DNase I (Aminsan, Iran). One microgram of each total RNA was reverse transcribed to cDNA using the first strand cDNA synthesis kit (YTA, Iran). Quantitative real‐time PCR was performed to assess gene expression by the StepOnePlus Real‐Time PCR System (ABI, USA) using qPCRBIO SyGreen Mix (PCR Biosystems, UK). The PCR parameters were as follows: initial denaturation (one cycle at 95°C for 2 min); 40 cycles of denaturation, annealing and amplification (95°C for 5 s, 60°C–64°C for 30 s); and the melting curve (starting at 65°C and gradually increasing to 95°C). Gene expressions of TNF‐α, IL‐6, NF‐κB, Sirt1 and Nrf2 were normalised to the levels of GAPDH, and expression differences were calculated according to the standard curve and efficiency (*E*) established for each primer set (2^−ΔΔCT^ formula). Specific primers are listed in Table 1.

**TABLE 1:** Sequences of forward and reverse primers used for real‐time quantitative PCR analyses.

Primer	Sequence	
TNF‐α	Forward	AGGGTCTGGGCCATAGAACT
	Reverse	CCACCACGCTCTTCTGTCTAC
IL‐6	Forward	AGACTTCCATCCAGTTGCCT
	Reverse	CATTTCCACGATTTCCCAGAGA´
NF‐κB	Forward	AGCCACAGAGATGGAGGAGTTG
	Reverse	GGATGTCAGCACCAGCCTTTAG
Sirt1	Forward	AGCTCCTTGGAGACTGCGAT´
	Reverse	ATGAAGAGGTGTTGGTGGCA
Nrf2	Forward	CACCATGGGAATGGACTTGGAGCTGCC
	Reverse	CTAGTTTTTCTTAACATCTGGCTTCTTAC

### 2.8 Statistical Analysis

All the data generated from the research were presented as the mean ± standard deviation (SD). The values obtained were examined statistically by applying one‐way ANOVA technique to confirm statistical differences between the means of each group. Tukey test was carried out to determine the significance of the difference of means. All graphs were plotted by using GraphPad Prism 5 software (GraphPad Software Inc., San Diego, CA, USA).

## 3 Results

### 3.1 Biochemical Analysis

Blood biochemical test indicators were determined and the results summarised in Table 2.

**TABLE 2:** Changes in blood biochemical parameters in different mice groups.

Treatment groups	Glucose levels (mg/dL)	BUN (mg/dL)	CRT (mg/dL)	AST (U/L)	ALT (U/L)	Albumin (g/dL)
Group I	101.8 ± 3.30	55.2 ± 9.31	0.418 ± 0.08	32.42 ± 1.29	39.3 ± 4.57	4.06 ± 0.43
Group II	222.8 ± 7.04^a^	79.2 ± 5.02^b^	0.554 ± 0.03^b^	76.9 ± 8.92^a^	87.2 ± 3.98^a^	2.94 ± 0.40^a^
Group III	182.5 ± 6.60	73.0 ± 11.27	0.5 ± 0.04	64.4 ± 10.01	66.35 ± 2.87	2.8 ± 0.12
Group IV	167 ± 10.42	64.8 ± 14.65	0.488 ± 0.05	37.8 ± 5.62	45.85 ± 4.21	2.84 ± 0.15
Group V	153 ± 8.40	60.6 ± 6.73	0.452 ± 0.02	34.32 ± 5.83	43.5 ± 33.73	3.3 ± 0.2
Group VI	105.5 ± 1.73^e^	40.2 ± 5.49^e^	0.432 ± 0.00^e^	34.04 ± 3.18^c^	35.3 ± 5.89^c^	4.12 ± 0.17^c^
Group VII	108.3 ± 4.57^d^	62.4 ± 5.94^d^	0.448 ± 0.26^d^	26.36 ± 4.26^d^	38.65 ± 7.88^d^	4.3 ± 0.20^d^

*Note:* The data were presented as mean ± SD, *n* = 5/group.

^a^ *p* < 0.001 versus control group.

^b^ *p* < 0.01 versus control group.

^c^ *p* < 0.001 versus diabetic group.

^d^ *p* < 0.001 versus diabetic group.

^e^ *p* < 0.001 versus diabetic group.

#### 3.1.1 Pharmacological Intervention and Their Effects on Levels of Blood Glucose

The blood glucose level in the diabetic group with alloxan (120 mg/kg/day) increased significantly compared to the control group (*p* < 0.001). Blood glucose levels in the treated diabetic mice with dimethyl fumarate (20, 40 and 80 mg/kg/day) were significantly reduced compared to the diabetic group (*p* < 0.001) (Table 2), the highest dose being the most effective. Also, the glucose level in diabetic mice decreased post administration of MET (200 mg/kg/day) (*p* < 0.001).

#### 3.1.2 Pharmacological Intervention and Their Effects on Levels of Blood Urea Nitrogen

Blood urea is an effective clinical diagnostic indicator for chronic and acute kidney disease [18]. Diabetic mice showed a significant rise in blood urea level compared to the control group (*p* < 0.01). Blood urea level in treated mice with dimethyl fumarate (20, 40 and 80 mg/kg/day) decreased significantly compared to the alloxan group (120 mg/kg/day) in a dose‐dependent manner. The blood urea nitrogen level in diabetic mice decreased post administration of MET (200 mg/kg/day) (*p* < 0.001).

#### 3.1.3 Pharmacological Intervention and Their Effects on Levels of Blood Creatinine

Blood creatinine level was significantly elevated in diabetic mice compared to normal control group (*p* < 0.01). Excess level of blood creatinine indicates impaired glomerular filtration rate and kidney dysfunction [4]. The level of creatinine in the treated mice with dimethyl fumarate (20, 40 and 80 mg/kg/day) was significantly reduced compared to the alloxan group (120 mg/kg/day). Treated diabetic mice with MET (200 mg/kg/day) also reduced the blood creatinine level compared to the diabetic group (*p* < 0.001).

#### 3.1.4 Pharmacological Intervention and Their Effects on AST and ALT Levels

Blood ALT and AST levels were significantly elevated in diabetic mice compared to the control group (*p* < 0.001). Dimethyl fumarate‐diabetic mice showed significantly diminished levels of blood ALT and AST (~50%, *p* < 0.001 vs. the diabetic group). MET administration (200 mg/kg/day) also reduced the blood AST and ALT levels compared to the diabetic group (*p* < 0.001).

#### 3.1.5 Pharmacological Intervention and Their Effects on Levels of Blood Albumin

Diabetic kidney failure can be diagnosed with a higher urine albumin and a lesser amount in the serum [19]. In the current study, a noteworthy decrease in blood albumin levels was confirmed in diabetic mice as compared to the control group (*p* < 0.001). Administering dimethyl fumarate (80 mg/kg/day) significantly increased blood albumin level compared to diabetic mice (*p* < 0.001). The level of albumin in the treated mice with MET (200 mg/kg/day) increased significantly compared to the diabetic group (*p* < 0.001).

### 3.2 Assessment of Oxidative Stress Markers

#### 3.2.1 Pharmacological Intervention and Their Effects on Kidney and Liver Protein Carbonyl Content

**FIGURE 1:**
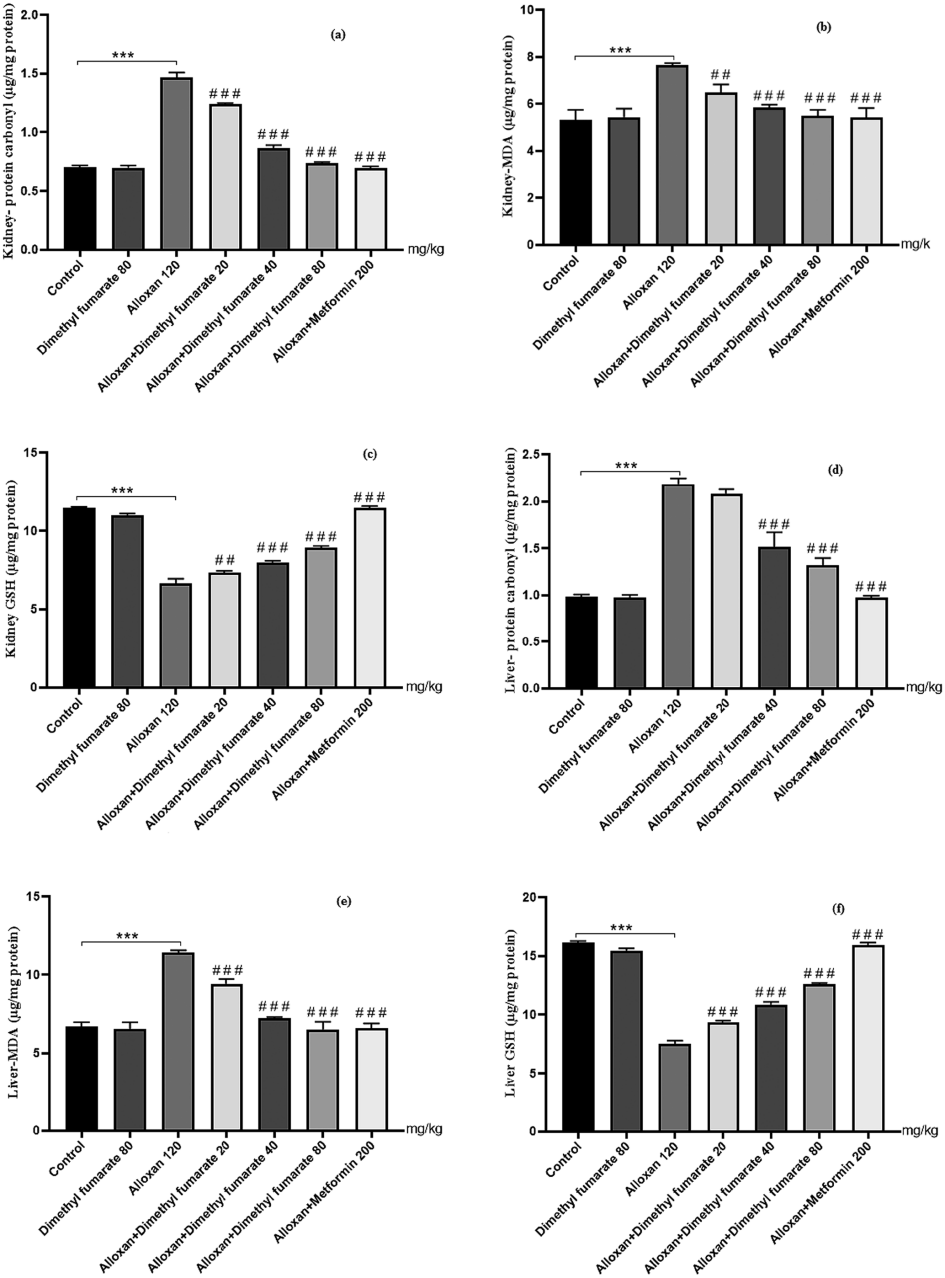
Effects of different doses of dimethyl fumarate (20, 40 and 80 mg/kg) on hepato‐renal oxidative biomarkers. Data are presented as mean ± SD (*n* = 5). ***p < 0.001 versus control group. ^###^ *p* < 0.001 versus diabetic group. ^##^ *p* < 0.001.

A subsequent growth in concentrations of protein carbonyl content was witnessed in alloxan‐induced diabetic mice when equated against the normal control group (Figure 1a,d, *p* < 0.001). Administrating dimethyl fumarate (20, 40 and 80 mg/kg/day) for 21 days decreased levels of protein carbonyl content compared to the diabetic group in a dose‐dependent manner. The highest dose showed maximum therapeutic effects (80 mg/kg/day) (*p* < 0.001). The protein carbonyl content in the treated mice with MET (200 mg/kg/day) decreased significantly compared to the diabetic group (*p* < 0.001).

#### 3.2.2 Pharmacological Intervention and Their Effects on Kidney and Liver MDA Content

The concentration of MDA in diabetic kidney and liver increased post induction of alloxan when equated against control group indicating augmented oxidative stress (Figure 1b,e, *p* < 0.001). Administration of dimethyl fumarate for 3 weeks at low (20 mg/kg/day), medium (40 mg/kg/day) and high (80 mg/kg/day) doses, po reduced the levels of hepato‐renal MDA compared to diabetic group in a dose‐dependent manner (*p* < 0.001). The MDA content in the treated mice with MET (200 mg/kg/day) decreased significantly compared to diabetic group (*p* < 0.001).

#### 3.2.3 Pharmacological Intervention and Their Effects on Kidney and Liver GSH Levels

A subsequent drop in concentrations of GSH levels was witnessed in alloxan‐induced diabetic mice when equated against the normal control group (*p* < 0.001). Administrating dimethyl fumarate for 21 days at low, medium and high doses amplified levels of GSH in a dose‐dependent manner compared to alloxan‐induced diabetic mice (*p* < 0.001). The highest dose (80 mg/kg/day) showed maximum therapeutic effects (Figure 1c,f). The GSH levels in the treated mice with MET (200 mg/kg/day) increased significantly compared to the diabetic group (*p* < 0.001).

### 3.3 Histopathological Observations

#### 3.3.1 Renal Histopathological Changes

**FIGURE 2:**
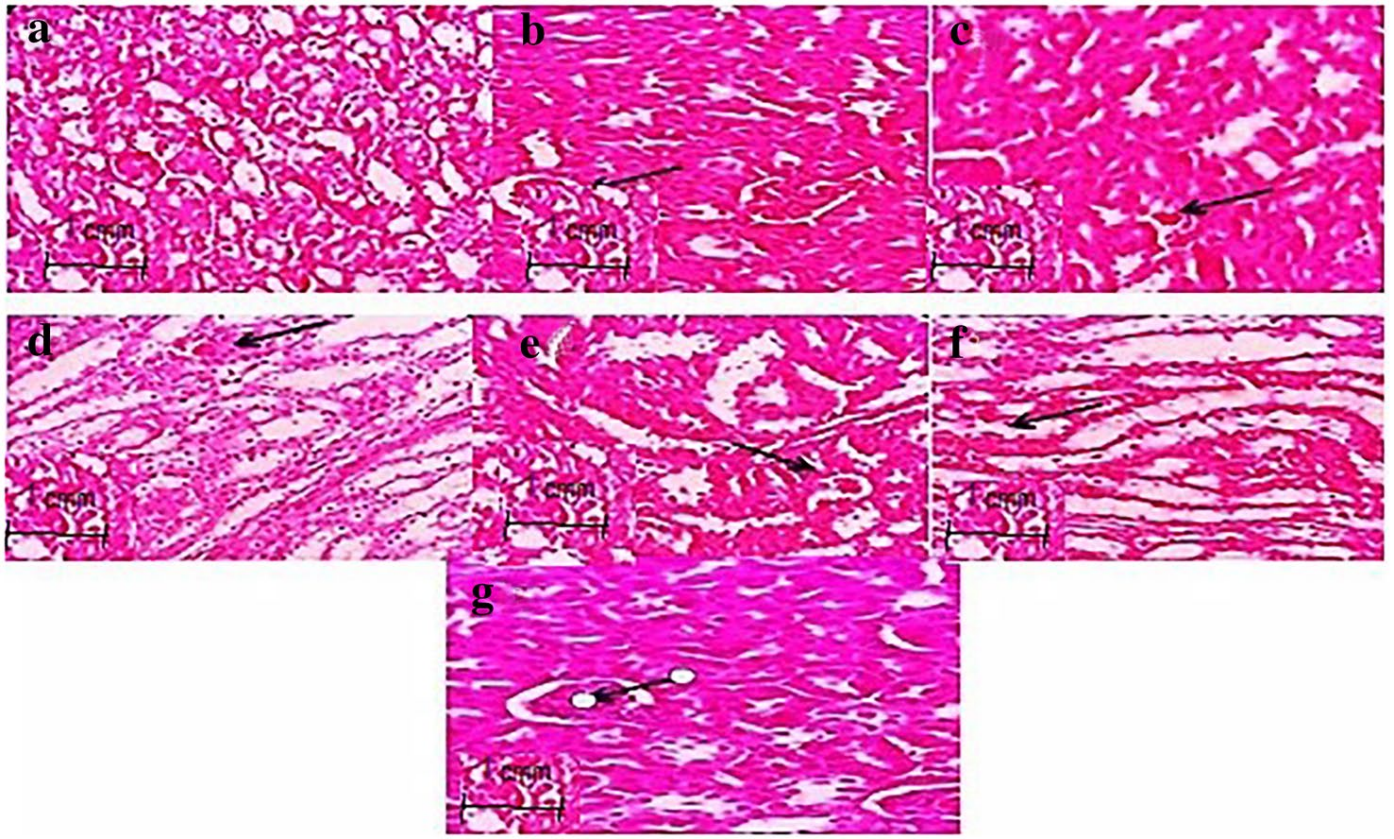
Effect of dimethyl fumarate on renal histologic changes in diabetic mice (H&E staining 40×): (a) control group, (b) alloxan group (120 mg/kg), (c) alloxan + dimethyl fumarate group (20 mg/kg), (d) alloxan + dimethyl fumarate group (40 mg/kg), (e) alloxan + dimethyl fumarate (80 mg/kg), (f) dimethyl fumarate (80 mg/kg), (g) alloxan + metformin (200 mg/kg). The figure is related to one sample out of three samples examined in each group (*n* = 1 per experimental group).

Histopathological examination of renal tissue was undergone by using haematoxylin–eosin staining (Figure 2). Control mice kidney section showing normal renal architecture (Figure 2a). As seen in the Figure 2, with alloxan injection, mild dilation and degeneration of tubules were observed in diabetic mice (purple and green dots, respectively) as compared with the normal control group (Figure 2b). On the other hand, the renal section of diabetic mice (120 mg/kg, ip) showed shrunken glomerular tufts, increase in Bowmans space, and dilation of proximal and distal convulated tubules with relatively higher number of mesangial cells. The renal section of diabetic mice post‐treatment with dimethyl fumarate for 21 consecutive days at medium doses (40 mg/kg/day, po) showed that the usual appearance and size of the glomerular capillaries were retained. The Bowman's capsule, proximal and distal tubules also improved in size and thickness. In the treated mice with dimethyl fumarate (80 mg/kg/day), we saw inflammation, dilation of tubules and their deformation (Figure 2f; purple, red and green dots).

#### 3.3.2 Hepatic Histopathological Changes

**FIGURE 3:**
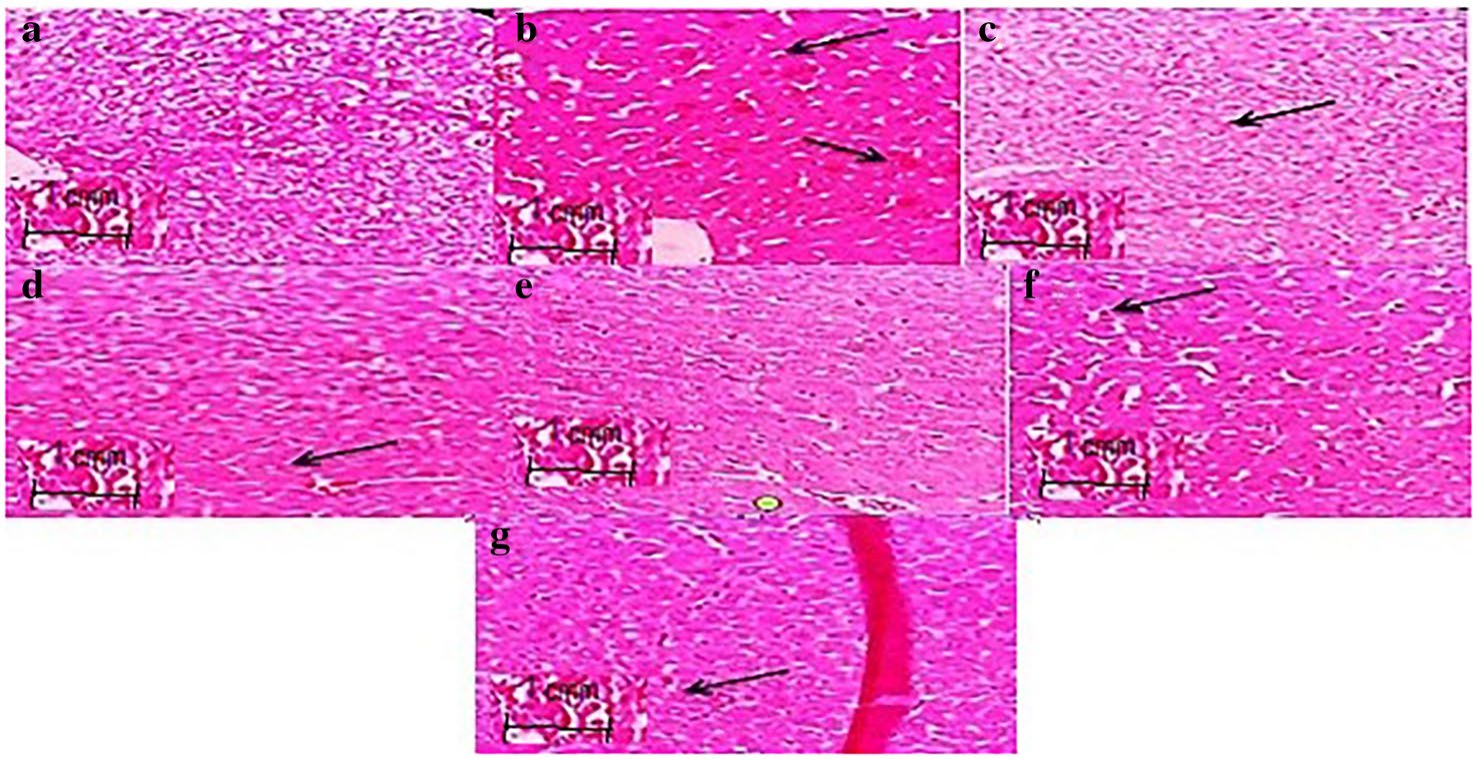
Effect of dimethyl fumarate on liver histologic changes in diabetic mice (H&E staining 40×): (A) control group, (B) alloxan group (120 mg/kg), (C) alloxan + dimethyl fumarate group (20 mg/kg), (D) alloxan + dimethyl fumarate group (40 mg/kg), (E) alloxan + dimethyl fumarate (80 mg/kg), (F) dimethyl fumarate (80 mg/kg), (G) alloxan + metformin (200 mg/kg). The figure is related to one sample out of three samples examined in each group (*n* = 1 per experimental group).

Photomicrograph of the control mice liver sections showed normal hepatic architectures with no inflammation which comprised normal central vein, hepatic cords, hepatocytes and portal area contents (bile duct, hepatic artery and vein) (Figure 3a). Liver sections of the alloxan‐diabetic mice showed mild hepatic injuries such as inflammation and enlargement of the bile ducts (Figure 3b). However, no signs of fibrosis or fatty liver were observed in this group. Liver sections taken from the treated mice at low and medium doses with dimethyl fumarate showed less bile duct dilatation compared to other groups (Figure 3c,d). In treated mice with alloxan + MET (200 mg/kg/day), severe hepatic tubules inflammation and dilation were observed (Figure 3g, purple and yellow dots, respectively).

### 3.4 Effect of Dimethyl Fumarate on Reno‐Hepato Inflammatory Genes Expression

#### 3.4.1 Renal Levels of Inflammatory Genes Expression

**FIGURE 4:**
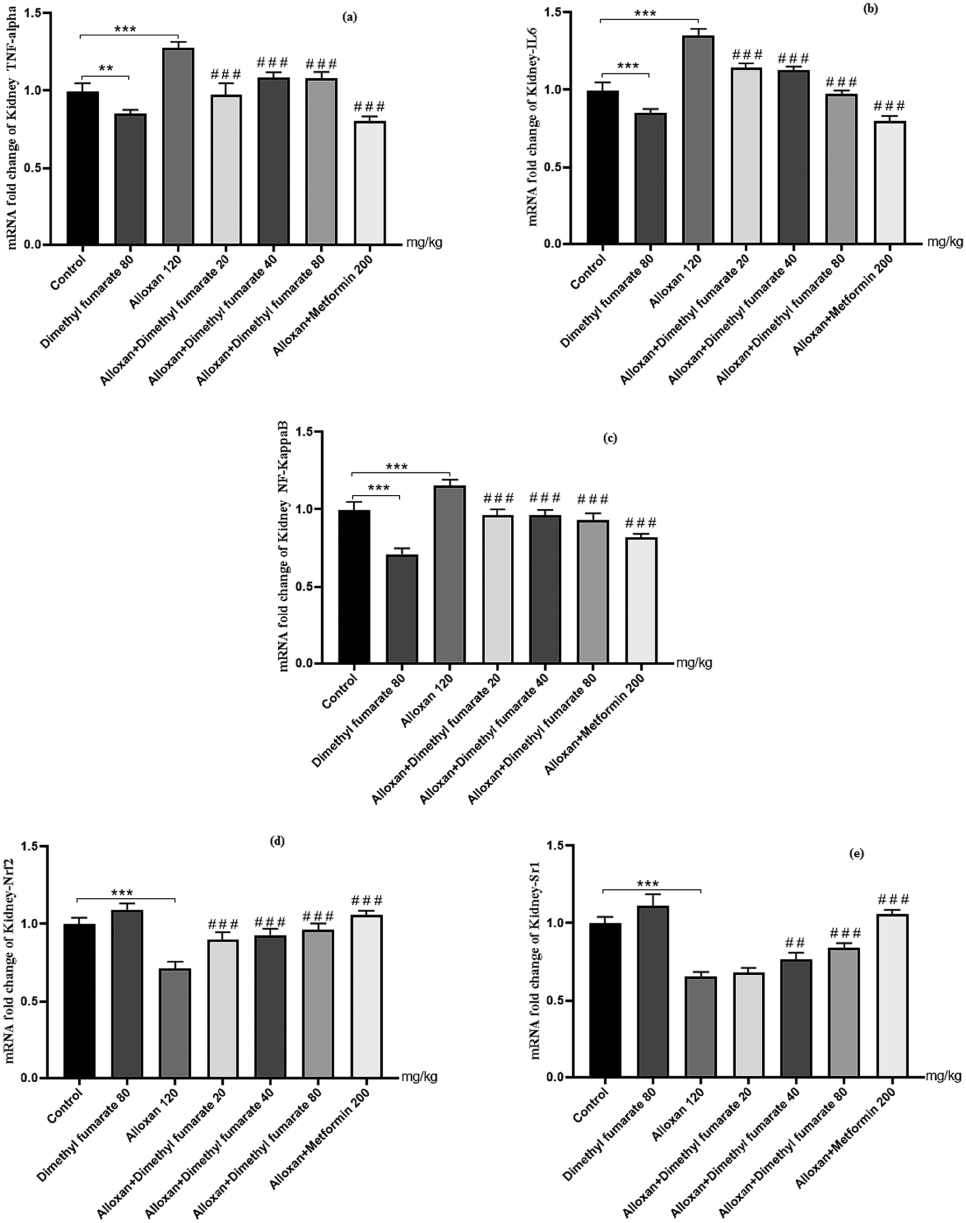
Effect of dimethyl fumarate on renal levels of inflammatory genes expression in diabetic mice. Data are presented as mean ± SD (*n* = 5). ***p < 0.001 versus control group. ^###^ *p* < 0.001 versus diabetic group. **p < 0.01 versus control group ^##^ *p* < 0.01 versus diabetic group.

The TNF‐α, IL‐6 and NF‐κB expression levels in diabetic mice renal increased post administration of alloxan compared to the normal control group (*p* < 0.001, Figure 4a–c). Administration of dimethyl fumarate (20, 40 and 80 mg/kg/day, po for 3 week) significantly diminished renal pro‐inflammatory cytokines expression in treated animals compared to the diabetic group (*p* < 0.001) in a dose‐dependent way. Meanwhile, the expression of Sirt1 and Nrf2 genes in the treated mice with dimethyl fumarate (80 mg/kg) was significantly higher compared to the diabetic group (*p* < 0.001, Figure 4d,e). A significant decrease in levels of TNF‐α, IL‐6 and NF‐κB expression was witnessed upon administration of MET (200 mg/kg/day) for three consecutive weeks when compared to the diabetic group (see Figure 4a–c). The renal Nrf2 and Sirt1 expression levels of diabetic mice increased post administration of MET (200 mg/kg/day, *p* < 0.001) compared to the diabetic group.

#### 3.4.2 Hepatic Levels of Inflammatory Genes Expression

**FIGURE 5:**
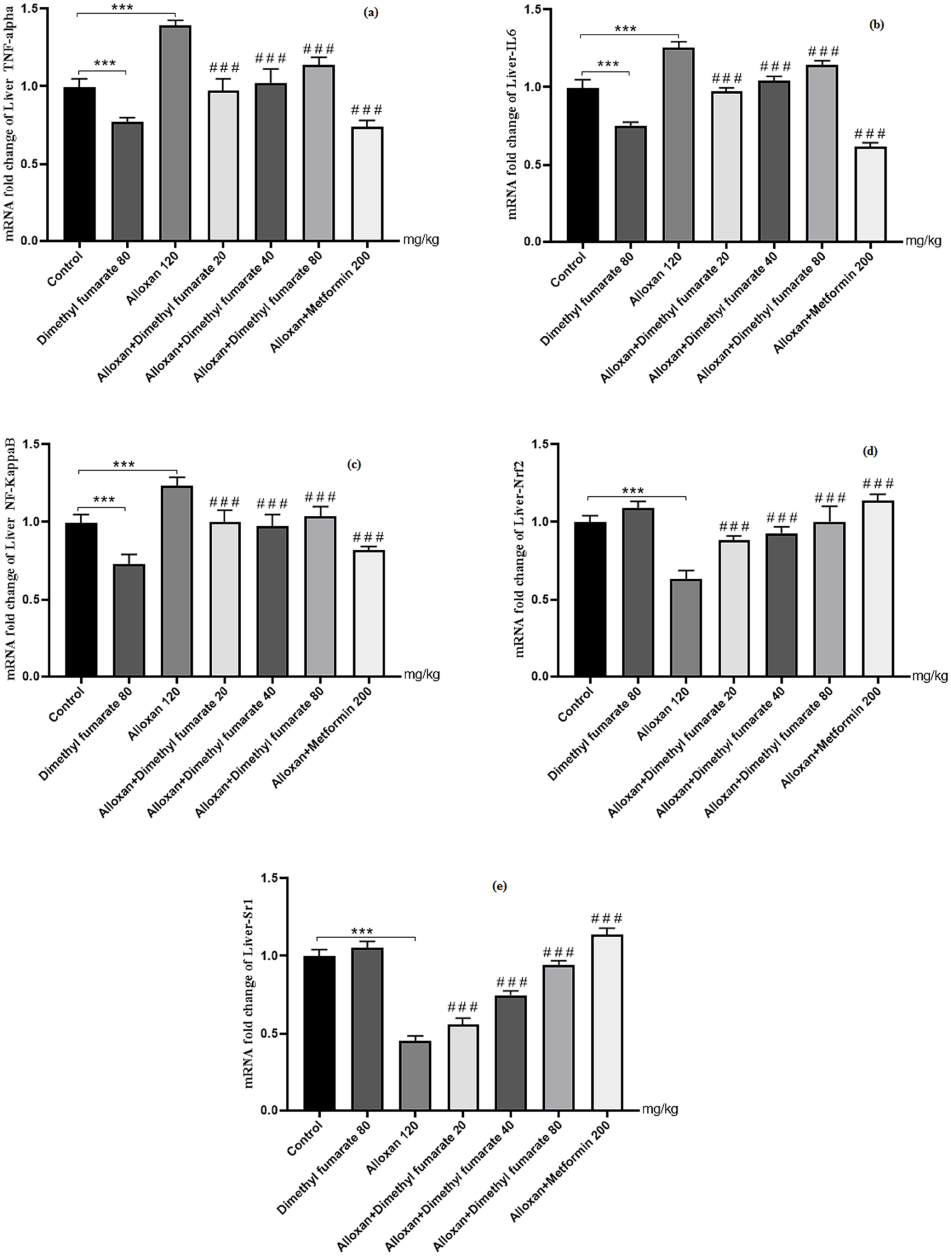
Effect of dimethyl fumarate on hepatic levels of inflammatory genes expression in diabetic mice. Data are presented as mean ± SD (*n* = 5). ***p < 0.001 versus control group. ^###^ *p* < 0.001 versus diabetic group.

**FIGURE 6:**
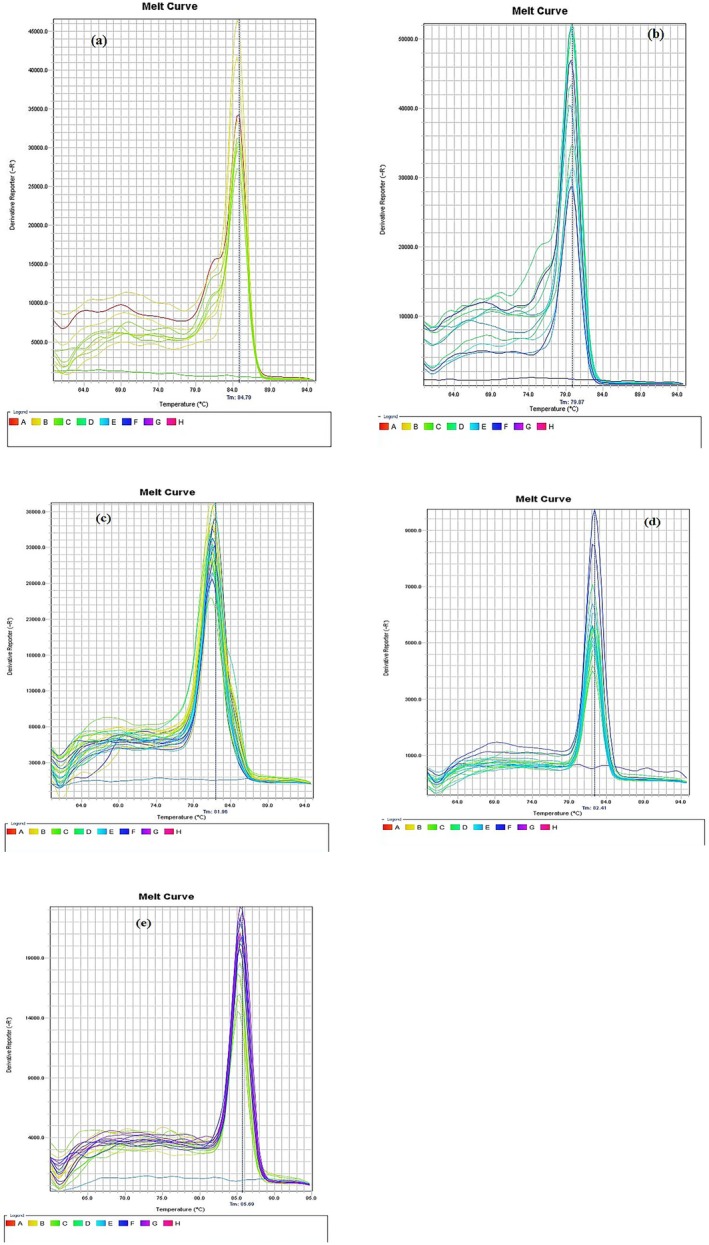
Melting curves of the TNF‐α (a), IL‐6 (b), Sirt1 (c), NrF2 (d), and NF‐KB (e) in a qPCR assay.

Dimethyl fumarate treatment markedly reduced TNF‐α, IL‐6 and NF‐κB (Figure 5a–c), increased levels of Nrf2 and Sirt1 in mice, probably emphasising its antioxidant potential reported previously [9] compared to diabetic values (*p* < 0.001). The Nrf2 and Sirt1 levels in the liver of diabetic mice decreased post administration of alloxan as compared to the normal control group (Figure 5d,e). The Nrf2 and Sirt1 levels improved on treatment with dimethyl fumarate (20, 40 and 80 mg/kg/day, po for 3 weeks) in comparison to alloxan‐induced diabetic animals; the highest dose showed maximum therapeutic effects (*p* < 0.001, Figure 5d,e). A significant decrease in levels of TNF‐α, IL‐6 and NF‐κB expression was witnessed upon administration of MET (200 mg/kg/day) for three consecutive weeks when compared to the diabetic group (see Figure 5a–c). The liver Nrf2 and Sirt1 expression levels of diabetic mice increased post administration of MET (200 mg/kg/day, *p* < 0.001) when compared to the diabetic group. Our findings suggest that dimethyl fumarate could potentially thwart diabetes‐induced liver tissue injury, likely via inactivation of pro‐inflammatory cytokines function, providing firm impetus for future repurposing of dimethyl fumarate in the management of hepatic complications. The melting curves of the inflammatory genes have been shown along with Sirt1 and Nrf2 genes expression in Figure 6.

## 4 Discussion

Chronic hyperglycaemia during diabetes through several metabolic disorders of AGEs and increased production of ROS leads to the onset and progression of dysfunction of the vital organs of the body, especially the kidneys and liver [5, 20]. In recent years, an association between the progression of diabetes and the incidence of hepato‐renal dysfunction, such as diabetic nephropathy, fatty liver disease, abnormal function of liver enzymes and acute liver failure has been observed [20]. So far, many evidences have been reported on the effective role of inflammation and oxidative stress in liver and kidney dysfunction during diabetes [10]. In the present study, the protective potential of dimethyl fumarate was investigated in the alloxan‐induced diabetic mouse model (as a highly cytotoxic glucose analogue) on two major organs involved in diabetes (liver and kidney). The obtained results showed that the mice treated with alloxan had hyperglycaemia and liver‐kidney damage caused by the increase of urea, creatinine, ALT, AST, MDA and carbonyl protein. Meanwhile, blood albumin and GSH levels decreased. Activation of different signalling pathways by ROS during diabetes causes liver and kidney dysfunction. The generated ROS and the progression of the oxidative stress process can facilitate liver‐kidney inflammation and the production of fibrosis. The latter can also contribute to a further increase in ROS production [9]. Also, the overproduction of ROS plays a critical role in the initiation and progression of diabetic kidney disease from the perspective of renal inflammation, affecting renal structure and function [4].

Dimethyl fumarate is a potent Nrf2 activator used for the clinical treatment of multiple sclerosis. The mechanism of action of dimethyl fumarate is unclear [11, 12]. Dimethyl fumarate inhibits the formation of pro‐inflammatory cytokines and NF‐κB signalling through its nuclear transduction pathway. This compound also has unique antioxidant properties [21]. It may help to manage blood sugar along with the administration of MET as a long‐standing drug effective in the treatment of diabetes, which is recommended by most experts as a first‐line treatment [22]. Few direct studies have been conducted on the effect of MET and dimethyl fumarate as anti‐diabetic drugs. Dimethyl fumarate inhibits the production of pro‐inflammatory cytokines and NF‐κB signalling by inhibiting its nuclear translocation. This drug also has unique antioxidant properties [21]. Despite its promising therapeutic effect on multiple sclerosis, the role of dimethyl fumarate in ameliorating diabetic liver and kidney dysfunction is poorly understood.

Pro‐inflammatory cytokines, such as TNF‐α, NF‐κB, IL6 expression is directly related to some diseases such as atherosclerosis, obesity, diabetes and cancer. Collectively, these findings suggest that dimethyl fumarate exerts its protective effects on the liver and kidney of diabetic rats, possibly through attenuating the ROS pro‐inflammatory cytokine pathway. The administration of dimethyl fumarate has a synergistic effect on the activity of SIRT1 and Nrf2, while also having an anti‐inflammatory effect. Possible mechanisms of dimethyl fumarate in restoring liver‐kidney function include suppressing activation of inflammatory genes expression through disruption of ROS‐NF‐kB‐dependent mediators and pathways. Based on these observations, dimethyl fumarate may be a promising drug for the prevention of hepato‐renal complications in diabetic patients [22, 23].

In this line, Lee et al. [24] stated that SIRT1 expression protects β‐cells against various toxic stresses such as oxidative stress and cytokines through the NF‐κB signalling suppression pathway. SIRT1 interacts with the insulin signalling pathway through different mechanisms. In another study, overexpression of SIRT1 in transgenic mice led to improved glucose tolerance due to reduced glucose output from the liver [25]. Metabolites such as free fatty acids along with TNF‐α cause large amounts of ROS to be produced in mitochondria. Therefore, reducing the oxidative capacity of mitochondria can induce insulin resistance through oxidative stress. The expression of the SIRT1 gene along with the overexpression of antioxidant enzymes through the acetylation pathway will lead to the reduction of liver damage [26].

Lone et al. [4] evaluated the protective potential of dimethyl fumarate during diabetic nephropathy caused by streptozotocin injection in mice. A significant increase in anti‐diabetic, protective and blood fat reduction effects was observed during the consumption of dimethyl fumarate. Treatment of diabetic rats with dimethyl fumarate for 28 days caused a significant decrease in blood glucose level, regulation of cholesterol triglyceride level with an increase in urine and serum parameters in addition to the antioxidant effect on the kidney. In general, the specific results of this study showed that dimethyl fumarate can be considered as an advanced option in the prevention of diabetic nephropathy [4].

Dimethylfumarate neutralises both the incompatible indicators of oxidative stress and inflammation through the regulation of Nrf2 gene expression and the activation of a set of downstream antioxidants. A review of the literature suggests that dimethyl fumarate activates Nrf2 after responses to oxidative stress as well as other possible events. Then, the level of inflammation from ROS‐NF‐κB signalling pathways and the expression of pro‐inflammatory cytokines in diabetic liver and kidney decreases [21, 27, 28].

Hu et al. [10] investigated the protective potential of dimethyl fumarate on possible myocardial tissue damage caused by diabetes through the activation pathway of Nrf2 function. They concluded that diabetic mice treated with dimethyl fumarate showed reduced oxidative stress, inflammation and fibrosis. Their results showed that dimethyl fumarate could potentially counteract diabetes‐induced myocardial tissue damage, possibly through activation of Nrf2 function [10].

In another study, Amin et al. [9], Ahmed and colleagues investigated the potential and key mechanisms of possible vascular protective effects of dimethyl fumarate on cardiac complications in diabetic rats. Based on their observations, dimethyl fumarate attenuates vascular remodelling and functional alterations in streptozotocin‐induced diabetic rats via several mechanisms, which mainly include suppression of NLRP3 inflammasome activation in diabetic aortas, possibly via impairing ROS‐TXNIP and/or ROS‐NF‐κB pathways [9].

During diabetes, the permeability and balance of hydrostatic and colloidal osmotic forces throughout the glomerular membrane of the kidney are disturbed [4, 19]. In our histopathology studies, the changes in the renal tissue have been observed in all experimental groups apart from the control mice on the basis of the typical histological architecture of the normal renal parenchyma (Figure 2) as observed previously by Lone et al. [4]. The results of our study showed several systemic disorders in liver and kidney cellular metabolism. The morphology of liver and kidney tissue in diabetic rats caused by alloxan injection underwent several vascular and inflammatory changes. A series of degenerative changes up to necrosis, tubular dilation, cell degeneration and interstitial eosinophilic infiltration were observed in the kidneys of diabetic mice.

In the liver tissue of diabetic mice, liver damage varying from vacuolar degeneration, dilation of tubes and oedema with the presence of newly formed non‐functional bile duct was observed. Our findings were in agreement with previously recorded by Lone et al. [4]. They evaluated the effect of dimethyl fumarate on kidney tissue changes for 28 consecutive days. Their results showed that the renal section of diabetic rats had a usual appearance and sizes of the glomerular capillaries were retained after treatment with dimethyl fumarate at 40 and 80 mg/kg/day [4].

Effect of dimethyl fumarate (25 mg/kg/day) on aortic histologic changes in streptozotocin‐induced diabetic rats was evaluated by Amin et al. [9]. Histopathological analysis of the aorta in diabetic rats showed the proliferation of fibrous tissue in the tunica media. These researchers concluded that structural changes were significantly reduced by dimethyl fumarate treatment. This trend may be related to the decrease in the level of Aortic transforming growth factor‐beta‐1 growth protein in diabetic rats treated with dimethyl fumarate compared to the untreated diabetic group [9]. Collectively, these findings indicated that dimethyl fumarate exerts favourable protective effects on diabetic rats, possibly through attenuating the ROS pro‐inflammatory cytokine pathway.

According to our study, alloxan made some decrease in the weight of animals and dimethylfumarate improved some of the weights. Also, we have some limitations in budget for immunohistochemistry complementary study; hopefully, in the near future, we will complete it.

Also there are some studies about the biocompatibility and safety profile of dimethyl fumarate. In one study, overall, 5124 patients received ≥ 1 dose of DMF. The mean (standard deviation [SD]) age at enrollment was 40.0 (11.2) years; 74% of patients were female. Patients received DMF for a mean (SD) duration of 31.0 (22.7) months. Primary reasons for discontinuation were AEs (*n* = 1237; 24%); the most common were gastrointestinal AEs (*n* = 469; 9%), blood and lymphatic disorders (*n* = 218; 4%), and vascular disorders (*n* = 200; 4%). SAEs occurred in 391 (8%) patients, most commonly infections and infestations (*n* = 102; 2%). Adjusted ARR declined by 90% (95% confidence interval [CI]: 90–91; *p* < 0.0001), from 0.81 (95% CI 0.79–0.84) 12 months before enrollment to 0.08 (95% CI 0.08–0.09) 6 years after enrollment. The estimated proportion of patients free from confirmed disability progression sustained over 48 weeks was 87.0% at month 60. Mean scores for physical and psychological impact, fatigue, health and productivity remained stable over 5 years [29].

## 5 Conclusions

Dimethyl fumarate treatment at 20, 40 and 80 mg/kg/day respectively for 21 days demonstrated an acceptable in blood glucose levels when compared with diabetic mice. A suitable downfall in blood creatinine, ALT, AST, protein carbonyl, MDA and blood urea levels was also witnessed compared to diabetic mice in a dose‐dependent manner.

Administering dimethyl fumarate (20, 40 and 80 mg/kg/day, po for 3 weeks) significantly increased the blood albumin concentration and the GSH content when compared against the diabetic group, the highest dose showing the best results. The histological features somewhat improved as shown by the normal size of glomerular capillaries along with less dilation of the bile ducts and the occurrence of inflammation when compared to diabetic mice. The protective effects of dimethyl fumarate were completely dependent on the expression of the genes evaluated in this study, and the expression of genes such as Sirt1 and Nrf2 caused the effective protection of dimethyl fumarate on liver and kidney function compared to diabetic mice.

The findings of the present study may indicate that dimethyl fumarate improves the restoration of liver‐kidney function in diabetic mice through several mechanisms such as decreasing cytokine and chemokine gene expression and increasing anti‐inflammatory responses. Our findings demonstrated that dimethyl fumarate may be repurposed for future clinical use in the management of hepato‐renal injuries and other complications of diabetes. There should be more studies in this field in the future.

## Author Contributions

All authors contributed to the study conception and design. Material preparation, data collection and analysis were performed by Parisa Saberi‐Hasanabadi, Dr. Ramin Ataee as supervisor and designer of the project, Fatemeh Shaki collaborated and led the oxidative stress experiment, Mohammad Karami as toxicological consultant, Mohammad Ranaee as pathological experiments designer and director and Abouzar Bagheri as technical labs specialist collaborated in biochemical experiments. All authors read and approved the final manuscript.

## Funding

This work was supported by a grant from Mazandaran University of Medical Sciences with grant number 11716.

## Ethics Statement

The animal experimentation protocols were conducted in accordance with the recommendations of the Mazandaran University of Medical Sciences Animal Ethical Committee (Code: IR.MAZUMS.4.REC.1401.11716).

## Conflicts of Interest

The authors declare no conflicts of interest.

## Data Availability

The data that support the findings of this study are available on request from the corresponding author. The data are not publicly available due to privacy or ethical restrictions.
